# Single red blood cell analysis reveals elevated hemoglobin in poikilocytes

**DOI:** 10.1117/1.JBO.25.1.015004

**Published:** 2020-01-23

**Authors:** Suet Man Tsui, Rafay Ahmed, Noreen Amjad, Irfan Ahmed, Jingwei Yang, Francis Manno, Ishan Barman, Wei-Chuan Shih, Condon Lau

**Affiliations:** a City University of Hong Kong, Department of Physics, Hong Kong SAR, China; b Sukkur IBA University, Department of Electrical Engineering, Sukkur, Pakistan; c University of Sydney, School of Biomedical Engineering, Faculty of Engineering, Sydney, New South Wales, Australia; d Johns Hopkins University, Department of Mechanical Engineering, Baltimore, Maryland, United States; e Johns Hopkins University, Department of Oncology, Baltimore, Maryland, United States; f Johns Hopkins University, Department of Radiology and Radiological Science, Baltimore, Maryland, United States; g University of Houston, Department of Electrical and Computer Engineering, Houston, Texas, United States

**Keywords:** poikilocytes, blood, red blood cells, hemoglobin, Raman spectroscopy

## Abstract

Abnormally shaped red blood cells (RBCs), called poikilocytes, can cause anemia. At present, the biochemical abnormalities in poikilocytes are not well understood. Normal RBCs and poikilocytes were analyzed using whole-blood and single-cell methods. Poikilocytes were induced in rat blood by intragastrically administering titanium dioxide (${\mathrm{TiO}}_{2}$) nanoparticles. Complete blood count and inductively coupled plasma mass spectrometry analyses were performed on whole-blood to measure average RBC morphology, blood hemoglobin (HGB), iron content, and other blood parameters. Follow-up confocal Raman spectroscopy was performed on single RBCs to analyze cell-type-specific HGB content. Two types of poikilocytes, acanthocytes and echinocytes, were observed in ${\mathrm{TiO}}_{2}$ blood samples, along with normal RBCs. Acanthocytes (diameter 7.7±0.5  μm) and echinocytes (7.6±0.6  μm) were microscopically larger (p<0.05) than normal RBCs (6.6±0.4  μm) found in control blood samples (no ${\mathrm{TiO}}_{2}$ administration). Similarly, mean corpuscular volume was higher (p<0.05) in ${\mathrm{TiO}}_{2}$ whole-blood (70.70±1.97  fl) than in control whole-blood (67.42±2.03  fl). Poikilocytes also had higher HGB content. Mean corpuscular hemoglobin was higher (p<0.05) in ${\mathrm{TiO}}_{2}$ whole-blood (21.84±0.75  pg) than in control whole-blood (20.8±0.32  pg). Iron content was higher (p<0.001) in ${\mathrm{TiO}}_{2}$ whole-blood (697.0±24.5  mg/l) than in control whole-blood (503.4±38.5  mg/l), which supports elevated HGB as iron is found in HGB. HGB-associated Raman bands at 1637, 1585, and $1372\;{\mathrm{cm}}^{-1}$ had higher (p<0.001) amplitudes in acanthocytes and echinocytes than in RBCs from control blood and normal RBCs from ${\mathrm{TiO}}_{2}$ blood. Further, the $1585\text{-}{\mathrm{cm}}^{-1}$ band had a lower (p<0.05) amplitude in normal RBCs from ${\mathrm{TiO}}_{2}$ versus control RBCs. This represents biochemical abnormalities in normal appearing RBCs. Overall, poikilocytes, especially acanthocytes, have elevated HGB.

## 1 Introduction

Red blood cells (RBCs) are a major component of blood. They are important for transporting oxygen and other gases in the lungs. RBC abnormalities may occur in different diseases, such as liver disease, elevated cholesterol, and diabetes.^1^^–^^3^ Abnormally shaped RBCs (poikilocytes) may cause anemia, which increases health risks and cognitive loss.^4^^,^^5^ Poikilocytes can also be the result of exposure to chemicals, such as the nanoparticle (NP) titanium dioxide (${\mathrm{TiO}}_{2}$).^6^^–^^8^ ${\mathrm{TiO}}_{2}$ is common and widely used in various consumer products, such as cosmetics, foods, paints, paper, and sunscreens. Of note, a recent study published in the *Journal of the American Medical Association* found that sunscreen ingredients entered the blood stream in large amounts.^9^ There is a clear and considerable need to study blood abnormalities such as poikilocytes.

Current methods for studying blood include, but are not limited to, the complete blood count (CBC), the blood glucose test, and the blood cholesterol test. Blood tests provide a range of information such as RBC and white blood cell (WBC) counts, hemoglobin (HGB) levels, along with cell size measurements such as mean corpuscular volume (MCV). However, the information from blood tests is usually averaged across the whole sample, such as all RBCs, which fails to capture intercellular heterogeneity. Single-cell analyses can complement blood tests by providing information on heterogeneity, which is important for research to advance basic understanding of blood disorders. Optical tweezers have been employed to manipulate single RBCs to investigate shear modulus^10^ and to investigate interactions between RBCs^11^ in the presence of NPs.^12^ Micropipette aspiration has been employed to measure elongation.^13^ Raman spectroscopy has been employed to analyze diabetic RBCs^14^ and ABO blood typing.^15^ These single-cell studies provide valuable information on RBC changes during poikilocytosis and other conditions. However, the biochemical composition of poikilocytes, especially at the single-cell level, is not well understood.

In this study, we combine whole-blood analyses, specifically CBC and inductively coupled plasma mass spectrometry (ICP-MS), with single-cell analysis by confocal Raman spectroscopy. ${\mathrm{TiO}}_{2}$ NPs of 5-nm size were used to induce poikilocytes, as related studies have shown that the NPs affect the vascular system and damage liver function.^7^^,^^8^^,^^16^^–^^18^ Raman spectroscopy is a vibrational spectroscopic method that provides information on the chemical bonds in the sample and is applicable to many fields, including biomedicine.^19^^,^^20^ There is an increasing number of studies using Raman spectroscopy to examine blood and biomolecules,^21^^–^^24^ including in the presence of NPs such as nanodiamonds.^25^ In our study, two groups of rat subjects, low- and high-dose groups, were administered ${\mathrm{TiO}}_{2}$. Control groups were administered only water. Whole-blood from the high-dose group had a poikilocyte fraction of ∼70% (of RBCs) and was analyzed by CBC and ICP-MS. A high poikilocyte fraction is needed for the whole-blood methods to differentiate poikilocytes from normal RBCs in control blood samples. Single RBCs (poikilocytes and normal RBCs) from the low-dose group, with much lower poikilocyte fraction (∼30%), and controls were analyzed by Raman spectroscopy to obtain cell-type-specific information.

## 2 Methods and Materials

### 2.1 Chemicals

The ${\mathrm{TiO}}_{2}$ NPs used in this study were purchased from Anhui Elite Industrial Co., Ltd. (Elt-NT05) and the grain size was 5 nm. The NPs are 99.9% pure and the specific surface area ranges from 150 to $300\;m^{2}/g$. There are trace amounts of iron (≤5  ppm). For preparing the ${\mathrm{TiO}}_{2}$ suspensions, NPs were first mixed with distilled water at a body weight-dependent concentration, and then the suspensions were treated by ultrasonic vibration for 30 min before intragastric administration.

### 2.2 Animal Subjects and Dosing

All aspects of this study were approved by the animal research ethics committees of the City University of Hong Kong, the University of Hong Kong, and the Department of Health of the Hong Kong Special Administrative Region. Adult male Sprague Dawley rats (N=20) weighing 250 to 260 g were used in this study and acquired from the Laboratory Animal Unit of the University of Hong Kong. Subjects were housed in standard cages with a temperature of 25°C, humidity of 60% to 70%, 12/12 hour light/dark cycle, and regular access to food and drinking water at the Laboratory Animal Research Unit of the City University of Hong Kong.

Subjects were separated into low-dose control (N=5), low-dose experimental (N=5), high-dose control (N=5), and high-dose experimental (N=5) groups. For the control groups, the subjects were intragastrically administered (gavage) with distilled water every two days for 20 days (low-dose) and every day for 60 days (high-dose). For the experimental groups, the subjects were gavaged with ${\mathrm{TiO}}_{2}$ suspension at a concentration of 200  mg/kg body weight every two days for 20 days (low-dose) and 250  mg/kg every day for 60 days (high-dose). During gavage, a subject was wrapped in a towel and the back of its neck was grabbed tight to open its mouth. Afterward, a plastic feeding tube was inserted into the stomach via the oral cavity. Next, 1 ml of water or ${\mathrm{TiO}}_{2}$ suspension was injected into the subject.

### 2.3 Blood Collection and Sample Preparation

**Fig. 1:**
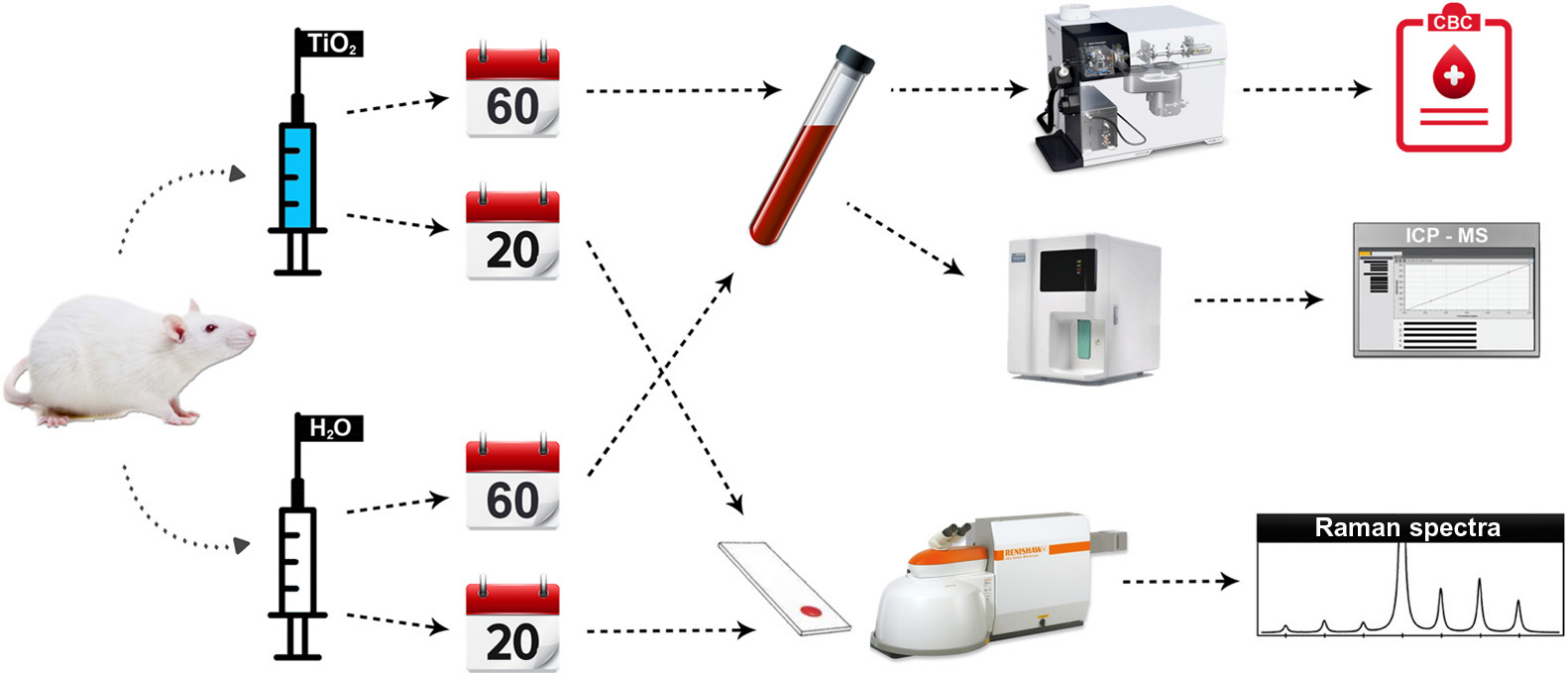
Experimental flowchart. Adult rats were administered ${\mathrm{TiO}}_{2}$ every other day by gavage at a dose of 200  mg/kg body weight for 20 days (low-dose, N=5) or 250  mg/kg every day for 60 days (high-dose, N=5). Low- and high-dose controls (N=5 each) were administered distilled water for equal durations. Whole-blood from high-dose subjects underwent a CBC and ICP-MS. Raman spectroscopy was performed on individual RBCs of low-dose subjects using 514-nm excitation and a confocal setup.

Twenty four hours after the last ${\mathrm{TiO}}_{2}$ injection, rats were anesthetized by pentobarbital (Alfasan International B.V.) and blood was collected by cardiac puncture. For ICP-MS and CBC performed on high-dose groups, 2.5 ml blood sample was collected. One and a half ml was placed in a sterile tube for ICP-MS and 1 ml was placed in ethylenediaminetetraacetic acid (EDTA) tubes (Jiangsu Yuli Medical Instrument Co., Ltd.) for CBC. For Raman spectroscopy performed on low-dose groups, 2 ml blood sample was collected and stored in EDTA tubes. Blood smears were prepared immediately after the sample was taken out from the EDTA tube. A drop of blood was placed from the syringe onto the glass slide (Shanghai Machinery Import and Export Company, Sail Brand 15 CAT. No. 7101). Afterward, a spreader slide was used to evenly distribute the blood on the glass slide. Figure 1 illustrates the experimental flow.

### 2.4 Trace Metal Quantification

Titanium (Ti) and iron (Fe) contents were measured from whole-blood samples based on Association of Analytical Communities 999.10 and determination by ICP-MS.^26^^–^^29^ Blood, nitric acid (${\mathrm{HNO}}_{3}$), and hydrogen peroxide ($H_{2}O_{2}$) were added to the digestion vessel and digested using a microwave digester at four powers 250, 630, 500, and 0 W for 3, 5, 22, and 15 min, respectively. Then the samples were transported to and analyzed using a PerkinElmer ELAN DRC II ICP-MS. Afterward, the results were quantified by an external calibration method using standard solutions. Calibration curves were drawn from calibration blanks at five standard points with different concentrations.

### 2.5 Complete Blood Count

Whole-blood samples were put inside the CBC counter and aspirated by the autosampler. The the samples were separated for the different tests and mixed with their respective reagents for cytochemical reactions. The peroxidase, RBC, basophil (BASO), reticulocyte, and HGB methods were used for the tests. RBCs, HGB, hematocrit (HCT), MCV, mean corpuscular hemoglobin (MCH), mean corpuscular hemoglobin concentration (MCHC), red cell distribution width (RDW), WBCs, neutrophils, lymphocytes, monocytes, eosinophils, BASO, platelet, and mean platelet volume were measured by a Siemens ADVIA 2120i cell counter.

### 2.6 Confocal Raman Spectroscopy

**Fig. 2:**
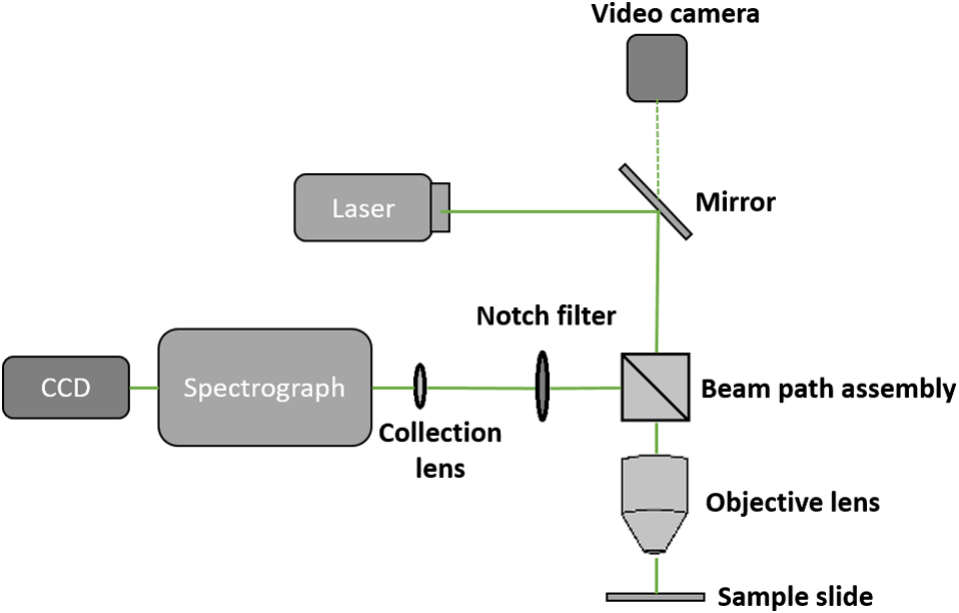
Schematic of Raman spectrometer with 514-nm laser and microscope (adapted from Raman Station Manual). CCD, charged coupled device (detector).

The confocal Raman system used in the study was the Renishaw inVia™ Qontor^®^ system from the United Kingdom.^30^^–^^33^ The Raman setup was first calibrated with a silicon reference (peak at $520\;{\mathrm{cm}}^{-1}$) before taking further measurements. After calibration, the blood smear samples were placed on a three-dimensional translation stage of the Raman microscope with a 50× objective from Nikon Japan. The target RBC was then selected under the microscope’s viewing system (500× magnification). With the confocal microscope setup, the vibrational signals from a micron-sized cell or particle can be measured. The standard mode laser spot size of the Raman system is ∼0.85  μm with the 50× objective. Figure 2 shows the Raman spectrometer schematic.

For spectrum acquisition, the target RBC was positioned under the center of the microscope’s field of view, which was also the position of the laser spot. After the target was placed in the right position, the light and camera were turned off. The mirror of the camera was also removed before the laser beam was turned on. Next, the beam spot was positioned at the center of the target RBC. Continuous laser excitation at 514 nm (green) was employed with the argon ion laser with 1800  l/mm grating for collection. A green laser was employed instead of a red or near-infrared laser to reduce the spot size. From Fig. S1 in the Supplementary Material, the Raman peaks were not obscured by the fluorescence background. The Raman shift range was recorded between 1800 and $600\;{\mathrm{cm}}^{-1}$ using 10-s exposure time, 10 accumulations, and laser power of ∼2  mW. Two RBCs (normal) were analyzed per low-dose control smear. Two normal-looking RBCs and four poikilocytes (two acanthocytes and two echinocytes) were analyzed per low-dose experimental smear.

**Fig. 3:**
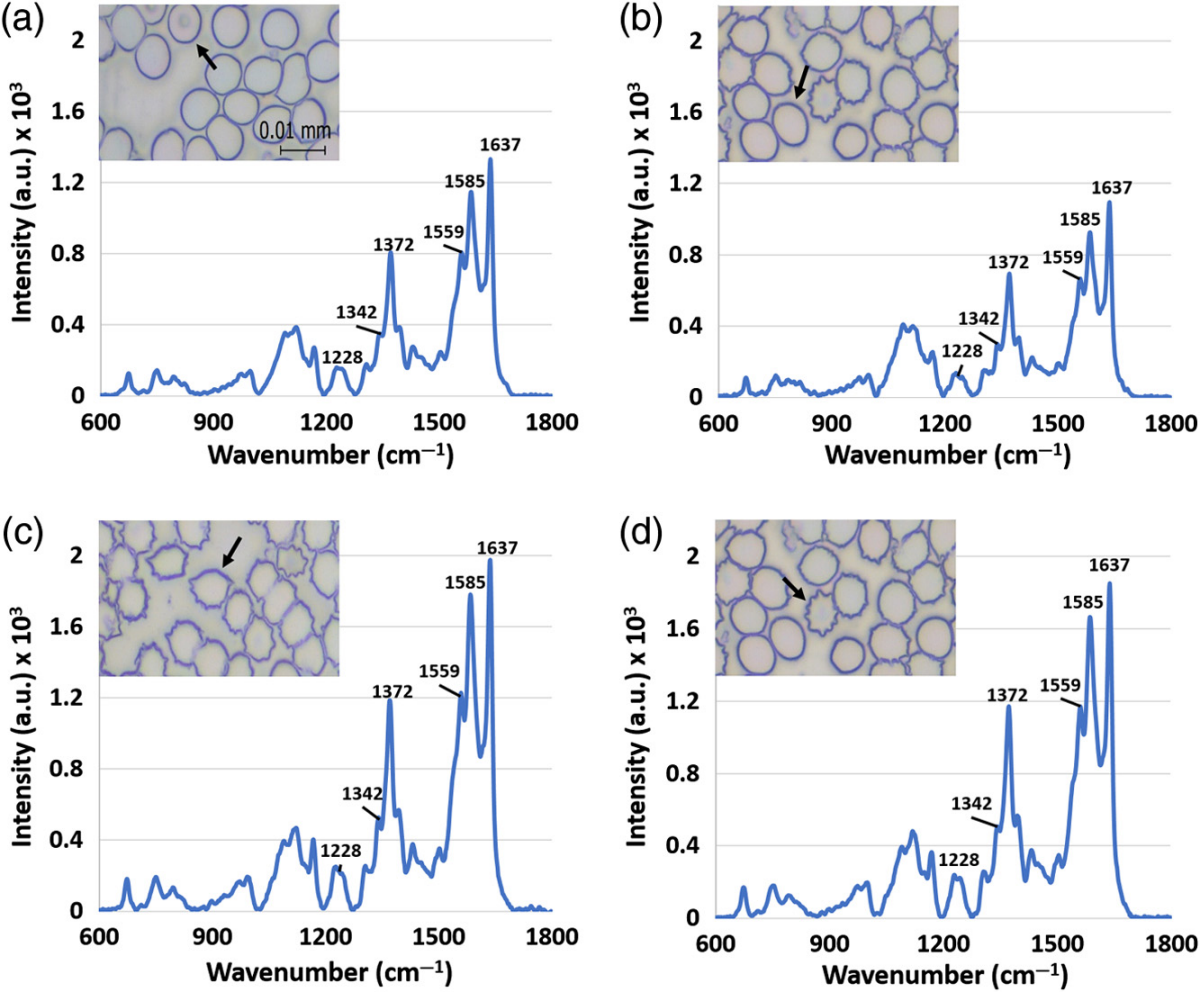
Representative RBCs (indicated by arrow) under 500× magnification and the corresponding Raman spectra acquired. (a) Normal RBC (n=10), (b) normal-looking RBC (n=10), (c) acanthocyte (n=10), and (d) echinocyte (n=10). The scale bar applies to all panels.

Spectrally analyzed RBCs from control subjects were termed normal. For RBCs from experimental subjects, those with normal appearance were grouped as normal-looking. Poikilocytes (acanthocytes and echinocytes) were observed. Note that normal-looking RBCs are not poikilocytes. Acanthocytes and echinocytes are both RBCs with an abnormal shape and small projections over the surface. The regularity of the spiculations can be used to distinguish acanthocytes from echinocytes.^34^ Echinocytes have a serrated outline with 10 to 30 small projections evenly distributed over the circumference, while acanthocytes have fewer spicules and the spicules are varying in length and thickness and are placed irregularly on the surface of the cell.^1^^,^^35^^,^^36^ The number and distribution of spicules on the cell were the main factors that were used in this study to distinguish between acanthocyte and echinocyte. Figure 3 shows the microscope images of the RBC types.

Each Raman spectrum was imported to Origin Pro 8.5 SR1 and Origin Pro 2017 for processing. Smoothing was performed using the Savitzky–Golay method, a local second-order polynomial regression with an eight-point window. Next, fifth-order polynomial fitting was used for background removal. After background removal, the wavenumber of the peaks of the spectra were found by determining the wavenumber with the highest intensity around the chosen peak. Peak heights were measured by fitting a Gaussian to peaks clearly visible on all spectra (1637, 1585, 1372, and $1168\;{\mathrm{cm}}^{-1}$, see Fig. 3) and recording the maximum. Peak heights were normalized by the integration of Raman signals at low wavenumbers ($<1100\;{\mathrm{cm}}^{-1}$, where HGB has few Raman bands^37^). The normalized height information was used for further statistical analysis. Table S1 in the Supplementary Material shows the Raman peak assignments for RBCs.^21^^,^^38^^,^^39^

The ratio of poikilocytes was determined by counting the number of poikilocytes and normal-looking RBCs in the blood smear under the field of view of the microscope. The diameters of the RBCs were measured and classified for different types (10 cells of each type per smear). Acanthocytes are not regular in shape and the largest length of the cell was marked as the diameter. The diameter data were used for further statistical analysis and compared with the MCV data from literature.

### 2.7 Statistical Analysis

A two-tailed t-test, one-way, or two-way analysis of variance (ANOVA) followed by Tukey’s HSD (honest significant difference) test were applied as appropriate for statistical analysis. The data were expressed as mean±standard deviation. Statistically significant difference, denoted as significant in the rest of this paper, was defined as p<0.05.

## 3 Results

### 3.1 TiO_2_ Elevates Blood Titanium

**Fig. 4:**
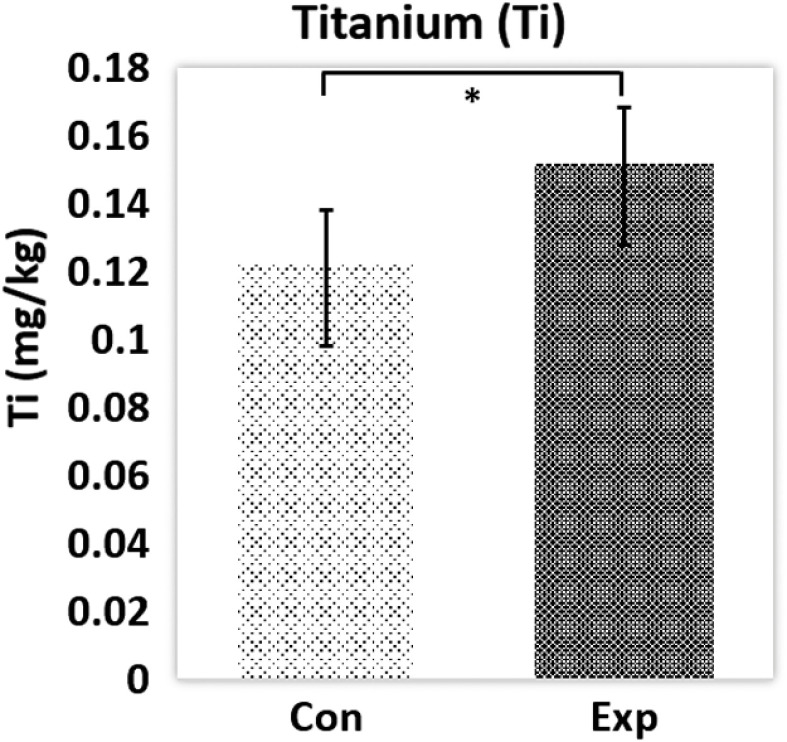
Blood titanium concentrations for high-dose control (Con, n=5) and experimental (Exp, n=5) groups measured by ICP-MS. Data are presented as mean and standard deviation. The * indicates p<0.05 by two-tailed t-test.

Titanium contents from the blood of the high-dose control and experimental groups were measured using ICP-MS (see Fig. 4). The mean titanium contents were 0.12±0.02  mg/l and 0.15±0.02  mg/l in control and experimental groups, respectively. The results show that blood titanium content in the high-dose experimental group was significantly higher than that in the control group (p<0.05). This confirms that the ${\mathrm{TiO}}_{2}$ NP treatment delivered titanium into the blood. Note that titanium has been previously reported at ∼0.1  mg/l levels in normal rat tissues.^40^^,^^41^ This likely comes from trace titanium in food and water.

### 3.2 Poikilocytes Are Larger Than Normal RBCs

Poikilocytes, abnormally shaped RBCs, were observed in the blood smear from the low-dose experimental group. Two types of poikilocytes (acanthocyte and echinocyte) were observed. Figure 3 shows four different types of RBCs from the blood smears: normal RBCs from controls [Fig. 3(a)], normal-looking RBCs [Fig. 3(b)], acanthocytes [Fig. 3(c)], and echinocytes [Fig. 3(d)] from experimental subjects under 500× magnification.

**Fig. 5:**
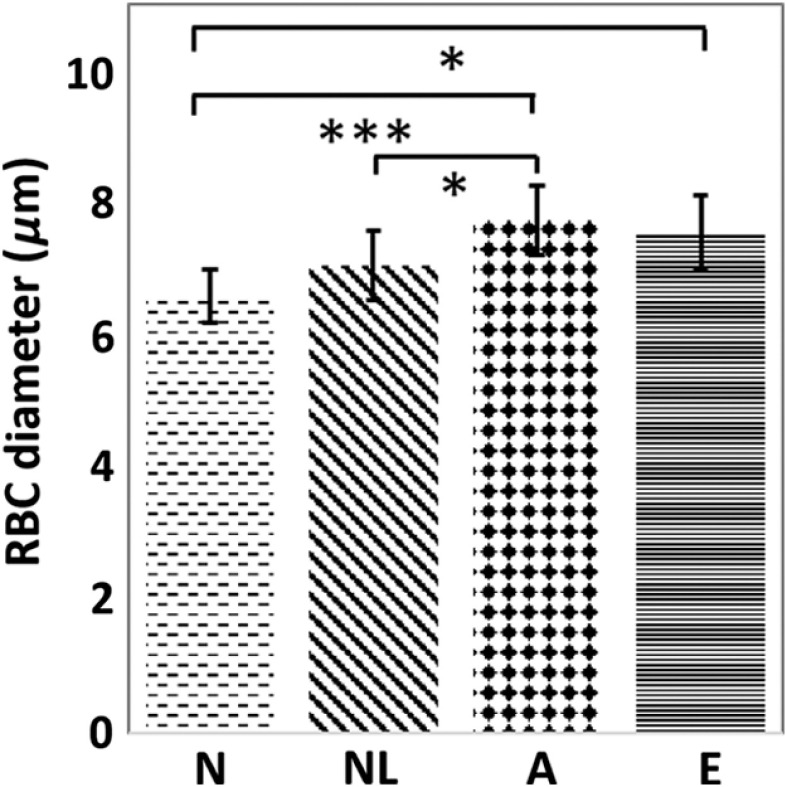
RBC diameters for normal RBCs (N), normal-looking RBCs (NL), acanthocytes (A), and echinocytes (E). Data are presented as mean and standard deviation. The * indicates p<0.05 and *** indicates p<0.001 by one-way ANOVA and Tukey’s HSD test.

The average diameters of normal, normal-looking RBCs, acanthocytes, and echinocytes were 6.6±0.4  μm, 7.1±0.5  μm, 7.7±0.5  μm, and 7.6±0.6  μm, respectively (Fig. 5). The diameters of acanthocytes and echinocytes were significantly larger than that of the normal RBCs with p<0.001 and p<0.05, respectively. The diameter of acanthocytes was larger than that of the normal-looking RBCs with p<0.05. For reference, normal RBCs in rats are 6 to 7  μm in diameter according to the literature.^42^

### 3.3 Blood with High Poikilocyte Ratio Has Elevated Hemoglobin

**Fig. 6:**
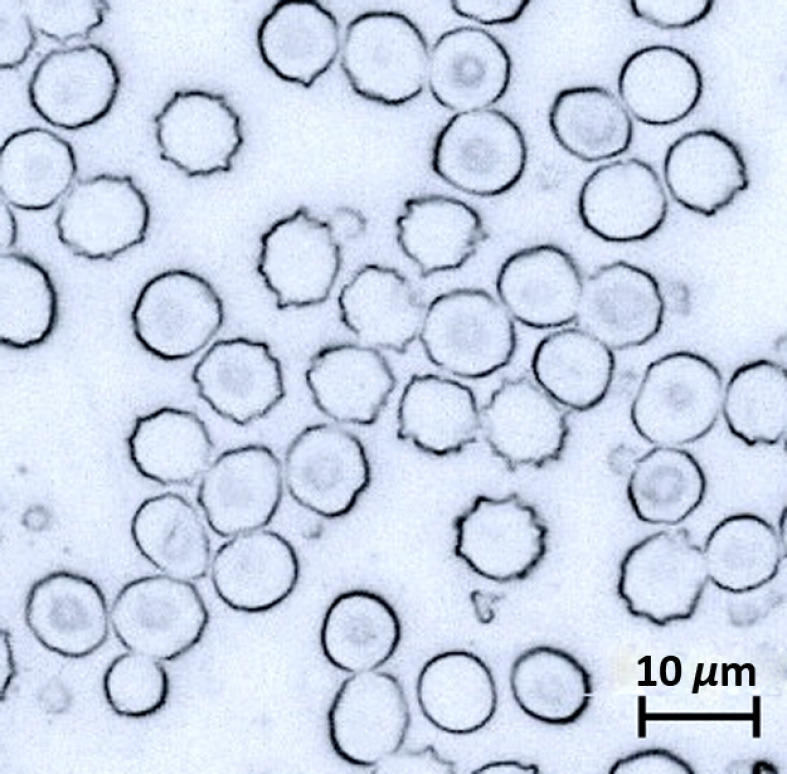
RBCs under 500× magnification for high-dose experimental group.

Many poikilocytes, ∼70% of RBCs, were observed in the blood smears from the high-dose experimental group (Fig. 6). In the low-dose group, the poikilocyte fraction was ∼30%. Both acanthocytes and echinocytes were observed. The high ratio of poikilocytes in the high-dose group facilitated using whole-blood CBC and ICP-MS measurements to analyze poikilocytes when compared with controls (no poikilocytes).

**Fig. 7:**
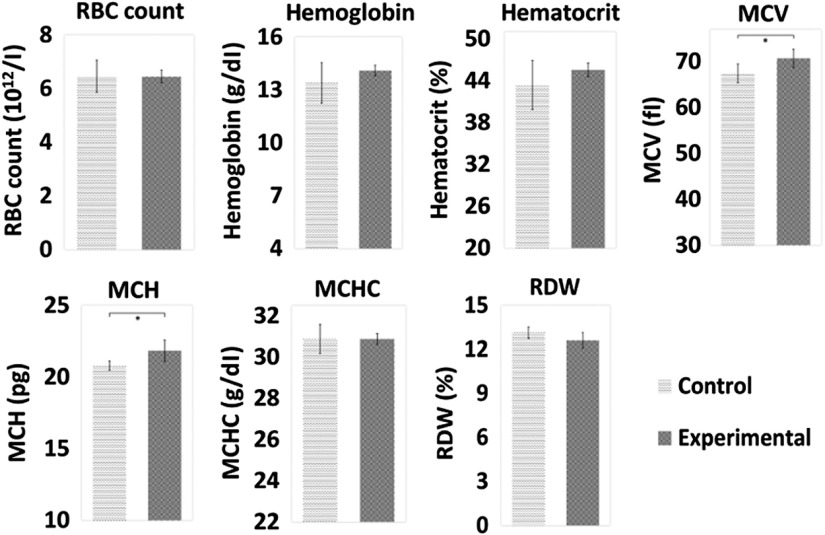
RBC count, HGB, HCT, MCV, MCH, MCHC, and RDW for high-dose control (n=5) and experimental groups (n=5). Data are presented as mean and standard deviation. The * indicates p<0.05 by one-way ANOVA and Tukey’s HSD test.

Figure 7 shows that both MCV, 70.70±1.97  fl, and MCH, 21.84±0.75  pg, in the high-dose experimental group were significantly higher than those in the control group, 67.42±2.03  fl and 20.8±0.32  pg, respectively (for both, p<0.05). For RBC count, HGB, HCT, MCHC, and RDW, the values were similar in both the control and experimental groups. Also, WBC and platelet measures were similar between the groups (see Figs. S2 and S3 in the Supplementary Material). Therefore, poikilocytes were on average larger and have more HGB than normal RBCs.

### 3.4 Blood with High Poikilocyte Ratio Has Elevated Iron

**Fig. 8:**
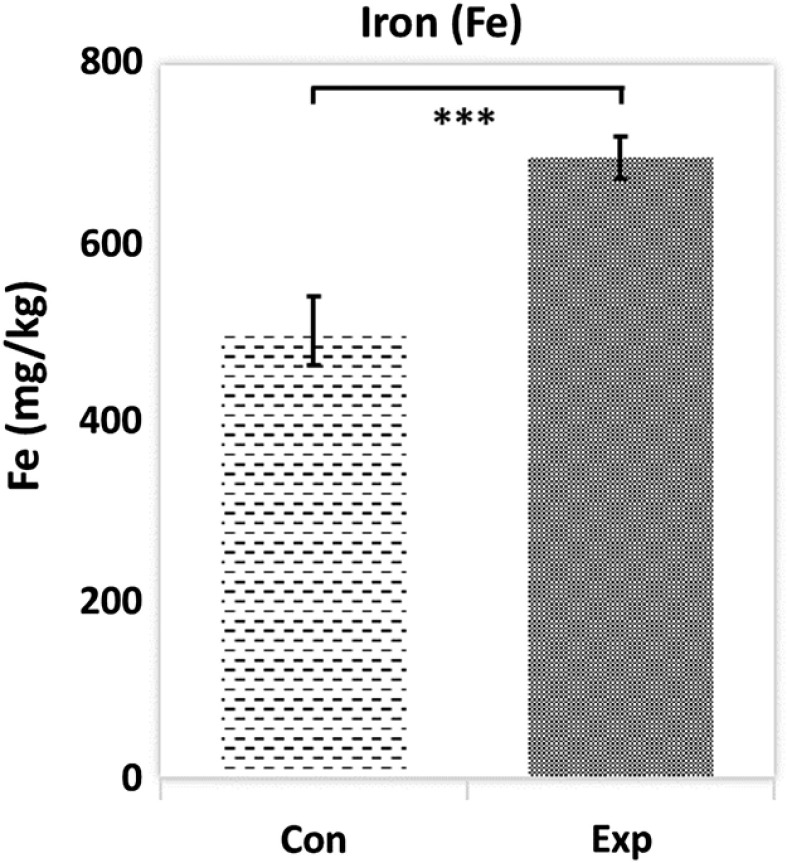
Blood iron concentration for high-dose control (n=5) and experimental groups (n=5) measured by ICP-MS. Data are presented as mean and standard deviation. The *** indicates p<0.001 by two-tailed t-test.

Whole-blood iron content of the high-dose control and experimental groups, measured by ICP-MS, were 503.4±38.5  mg/l in controls and 697.0±24.5  mg/l in experimental group (Fig. 8). The difference is statistically significant (p<0.001). Therefore, there is more iron in the blood during poikilocytosis. Iron is a key element in the HGB protein. Thus, ICP-MS corroborates the CBC findings of elevated HGB in poikilocytes.

### 3.5 Single-Cell Analysis Confirms Elevated Hemoglobin in Poikilocytes

To more precisely analyze poikilocytes and complement the above whole-blood analyses, individual RBCs from low-dose experimental and control groups were analyzed by Raman spectroscopy. For all four types of RBCs (normal RBC, normal-looking RBC, acanthocytes, and echinocytes), the Raman spectra were similar (Fig. 3). The bands were distributed over the range of 673 to $1637\;{\mathrm{cm}}^{-1}$. Raman spectra of RBCs can be separated into four different regions according to their corresponding vibration of different parts of the chemical structure.^21^ In the region of porphyrin in-plane vibrational modes, the peaks 1637, 1585, 1559, and $1431\;{\mathrm{cm}}^{-1}$ were observed. The bands 1627, 1604, and $1547\;{\mathrm{cm}}^{-1}$ were not observed. For the region of pyrrole ring stretching, the bands 1395, 1372, 1342, and $1306\;{\mathrm{cm}}^{-1}$ were observed. For the methane CH deformation region, 1245 and $1228\;{\mathrm{cm}}^{-1}$ were observed. For the low-wavenumber region, the bands 1166, 1132, 1090, 998, 975, 753, and $673\;{\mathrm{cm}}^{-1}$ were observed. The spectra of poikilocytes and normal-looking cells in the low- and high-dose groups were compared and show no big differences between the two doses (see Fig. S4 in the Supplementary Material).

**Fig. 9:**
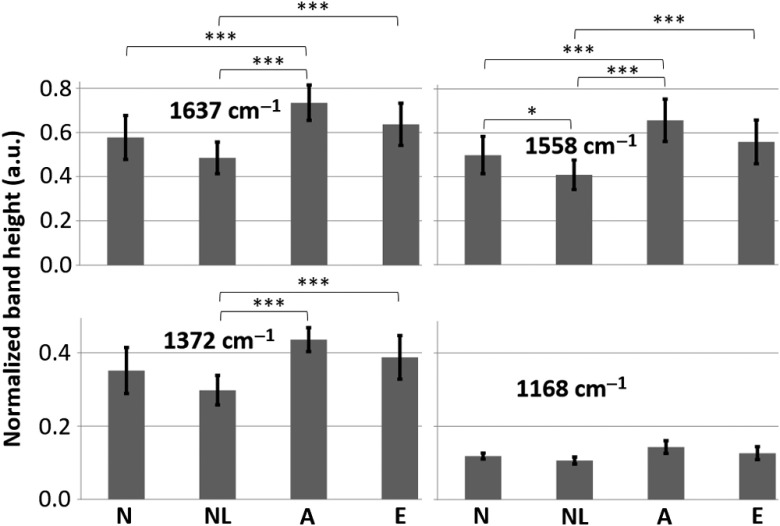
Raman band heights normalized by low wavenumber signal (see Sec. 2 for details) for normal RBCs (N), normal-looking RBCs (NL), acanthocytes (A), and echinocytes (E) at different wavenumbers. Data are presented as mean and standard deviation. The * and *** indicate p<0.05 and 0.001, respectively, by two-way ANOVA and Tukey’s HSD test.

For Raman spectra of acanthocytes, bands between 1650 and $1500\;{\mathrm{cm}}^{-1}$ and around 1400 and $1200\;{\mathrm{cm}}^{-1}$ were larger than that of other types of RBCs. Note that these bands were associated with oxygenated HGB Raman scattering.^37^ For quantitative analysis (see Fig. 9), the bands 1637 and $1585\;{\mathrm{cm}}^{-1}$, in the porphyrin in-plane and pyrrole ring regions, were considerably higher (after normalization) in acanthocytes than normal and normal-looking RBCs (p<0.001). For these two bands, echinocytes were higher than normal-looking RBCs (p<0.001). The $1372\text{-}{\mathrm{cm}}^{-1}$ band, in the pyrrole ring region, was higher in acanthocytes and echinocytes than in normal-looking RBCs (p<0.001). Interestingly, the $1585\text{-}{\mathrm{cm}}^{-1}$ band was higher in normal than in normal-looking RBCs (p<0.05). This may represent biochemical abnormalities in normal-looking RBCs in the absence of gross morphological abnormalities. Since HGB and RBCs in this study were oxygenated, poikilocytes likely contain more HGB than normal RBCs. In agreement with the band analysis, combined principal components and linear discriminant analysis of the Raman spectra show that normal and normal-looking spectra group together, while acanthocyte and echinocyte spectra group together (see Fig. S5 in the Supplementary Material).

## 4 Discussion

The increased blood titanium content of the ${\mathrm{TiO}}_{2}$ experimental group compared with the control group measured by ICP-MS indicates that titanium NPs were transported from the digestive system into the blood. Acanthocytes and echinocytes, two types of poikilocytes, were observed under microscope from ${\mathrm{TiO}}_{2}$ subjects and were on average larger than normal-looking RBCs. The CBC further showed that poikilocytes had more HGB than normal RBCs. Similarly, blood iron content was increased in the ${\mathrm{TiO}}_{2}$ group compared with the control group. From single RBC Raman spectroscopy, the intensities of HGB-associated bands at 1637 and $1585\;{\mathrm{cm}}^{-1}$ were larger in acanthocytes than in normal and normal-looking RBCs. These bands were also larger in echinocytes than in normal-looking RBCs. The intensity of the $1342\text{ -}{\mathrm{cm}}^{-1}$ band was larger in acanthocytes and echinocytes than in normal-looking RBCs and that of the $1585\text{-}{\mathrm{cm}}^{-1}$ band was larger in normal than in normal-looking RBCs.

### 4.1 Poikilocytes Are Enlarged

Acanthocytes and echinocytes in the low ${\mathrm{TiO}}_{2}$ dose group had larger diameters than normal RBCs in control subjects (Fig. 5), which was 6 to 7  μm. Grissa et al.^6^ and Duan et al.^7^ reported that the MCV of RBCs treated with ${\mathrm{TiO}}_{2}$ increased with increasing ${\mathrm{TiO}}_{2}$ dose and was proportional to the dose administrated. Average size increase in poikilocytosis was also observed in the high-dose group by the MCV of RBCs measurement in the CBC (Fig. 7).

### 4.2 Poikilocytes Have Elevated Hemoglobin

CBC and many traditional blood tests analyze a blood sample containing many cell types instead of single cells or a single cell type. Therefore, a high ${\mathrm{TiO}}_{2}$ dose was used to produce many poikilocytes. In our study, ∼70% of RBCs were poikilocytes in the high-dose experimental group (Fig. 6). From the CBC results, the MCH of RBCs after treatment with ${\mathrm{TiO}}_{2}$ was significantly higher than that of control subjects (Fig. 7). Therefore, RBCs in poikilocytosis have more HGB.

The iron content of blood from the high-dose experimental group was measured by ICP-MS and found to be higher than that of the control group (Fig. 8). Iron is an important element for blood production and about 73% of body iron is found in HGB.^43^ Therefore, blood iron content is closely related to the HGB level. This result further supports the CBC results of increased hemoglobin content in poikilocytes.

Follow-up analysis of individual RBCs with Raman spectroscopy (Fig. 9) showed that the bands in acanthocytes, especially 1637, 1585, and $1372\;{\mathrm{cm}}^{-1}$, were significantly higher than those of normal-looking RBCs. The band of $1637\;{\mathrm{cm}}^{-1}$ assigned to $v_{10}$ is due to translocation of the iron ion out of the heme plane during oxygenation. Therefore, oxygenation causes an increase in the intensity of this band.^44^ The $1585\text{-}{\mathrm{cm}}^{-1}$ band assigned to $v_{37}$ is larger in the oxygenated state than in the deoxygenated state in RBCs.^44^ The $1559\text{-}{\mathrm{cm}}^{-1}$ band assigned to $v_{2}$ only appears in RBCs in arterioles and is absent in venules.^24^ The $1372\text{-}{\mathrm{cm}}^{-1}$ band is the so-called oxidation state marker and appears in the spectra of RBCs in arterioles but is absent in venules.^24^ The $1228\text{-}{\mathrm{cm}}^{-1}$ band assigned to $v_{13}$ or $v_{42}$ becomes more intense as the cell becomes oxygenated.^21^ Based on this data, poikilocytes, especially acanthocytes, have higher HGB content than normal and normal-looking cells, as the intensities of the above bands were proportional to oxygenation.

### 4.3 Mechanisms of TiO_2_-Induced Poikilocytosis

In this study, we observed that exposure to ${\mathrm{TiO}}_{2}$ NPs leads to the formation of acanthocytes and echinocytes, two forms of poikilocytes. Acanthocytes result from alterations in RBC membrane lipids and proteins and can occur in patients with liver dysfunction, postsplenectomy, and other conditions.^35^^,^^45^ A resulting consequence of having acanthocytes in the blood is that they are vulnerable to being destroyed by the spleen, leading to anemia. In echinocytes, the mechanism of membrane alteration is different. Surface receptors on the RBC bind with cholesterol (do not integrate with the membrane), inducing the shape changes.^46^ Echinocytes may be found in patients with liver dysfunction. ${\mathrm{TiO}}_{2}$ NPs at the doses employed in this study are known to affect liver function and that of other organs such as the spleen.^7^^,^^18^ As acanthocyte and echinocyte formation are both closely related to liver (and spleen) dysfunction, this is the likely mechanism through which ${\mathrm{TiO}}_{2}$ induces poikilocytes.

### 4.4 Health Effects

Poikilocytes contain more HGB compared with normal and normal-looking RBCs. Excessive blood oxygen levels may cause hyperoxia, which is excessive oxygen in body tissues. Oxygen toxicity may cause nausea, dizziness, abnormal sensations, headache, disorientation, and lightheadedness.^47^ ${\mathrm{TiO}}_{2}$-treated subjects had higher blood iron level, which may elevate the risk of cardiovascular disease as high stored iron levels accelerate oxidation of cholesterol and damage the arteries.^48^

### 4.5 Technical Considerations

For this study, the CBC analysis showed similar MCHC between control and experimental groups. However, single-cell Raman spectroscopy demonstrated increased amplitude of HGB-related vibrational bands. Considering the laser spot size is considerably smaller than an RBC, this likely indicates increased HGB concentration in poikilocytes (rather than a larger cell with similar HGB concentration). In the authors’ opinion, the Raman observations are likely more representative in this case as CBC has to average across a large number of cell types. Such single-cell analysis is particularly important in the mechanistic elucidation of biological functions, due to the emerging consensus of cellular heterogeneity even within an isogenic cell population.^49^^,^^50^ Hence, single-cell Raman spectroscopy offers a key probe with spatial resolution that can overcome the limitations of stochastic averages computed from bulk measurements in heterogeneous populations. Ultimately, single-cell analysis can better delineate signaling pathways and networks, focus on specific populations aiding in differentiation of normal cells and poikilocytes, and suggest targeted therapeutic interventions.

The peaks observed due to the green, 514-nm laser applied to excite Raman scattering could be slightly different than other laser wavelengths (i.e., 633 nm^21^). Furthermore, the cells were placed on a glass slide for Raman spectroscopy. The glass background spectrum is shown in Fig. S1 in the Supplementary Material and compensated for as part of the background subtraction. It is worth noting that the location of the laser spot on different parts of an RBC will affect the Raman signal as the physical structure of an RBC is not homogeneous. In the spectra of normal RBC, three bands were missing: 1627, 1604, and $1547{\mathrm{cm}}^{-1}$, compared with the results of Wood and McNaughton.^21^ These bands were assigned to $v_{c═c}$, $v_{19}$, and $v_{11}$, respectively. These missing bands were likely merged with adjacent bands as the spectral resolution of our system was limited to $4\;{\mathrm{cm}}^{-1}$.

## 5 Conclusions

This study measured blood from rats treated with ${\mathrm{TiO}}_{2}$ NPs to analyze differences between poikilocytes and normal RBCs. CBC and ICP-MS measurements from poikilocytes were larger and carried more HGB than normal RBCs. Follow-up Raman spectroscopy analysis of single RBCs confirmed that poikilocytes, especially acanthocytes, carried more HGB. Further, single-cell Raman spectroscopy is able to study the biochemical differences between RBC types and advance basic hematologic understanding acquired primarily by whole-blood methods.
