# Supplementary material for: Single red blood cell analysis reveals elevated hemoglobin in poikilocytes
Source: J Biomed Opt. 2020 Jan 23;25(1):015004. doi: 10.1117/1.JBO.25.1.015004 (PMC6976897; doi:10.1117/1.JBO.25.1.015004)
Supplement: Supplementary file 1 [file JBO_025_015004_SD001.pdf]

Supplementary information

Raman peak assignments

Table S1 shows the Raman spectra peak assignments. For the range between 1650-1500 cm^{-1}, the peaks were mainly due to porphyrin in-plane vibrational modes. The most prominent bands appearing at 1637, 1627, 1604, 1585, 1559, 1547 and 1431 cm^{-1} were assigned to mode numbers v_{10}, $v_{c=c}$, v_{19}, v_{37}, v_2, v_{11} and v_{28}, respectively. For the range between 1400 and 1300 cm^{-1}, the peaks belonged to the region of pyrrole ring stretching. The 1395, 1372, 1342 and 1306 cm^{-1} bands were assigned to v_{20}, v_4, v_{41} and v_{21}, respectively. The peaks from 1300-1200 cm^{-1} belonged to the region of methane C–H deformation. The 1245 and 1228 cm^{-1} bands were assigned to v_{13} and v_{42}, respectively. The peaks ranged between 1200 – 600 cm^{-1} belonged to the low-wave number region. The 1166, 1132, 1090, 998, 975, 753 and 673 cm^{-1} bands were assigned to v_{30}, v_{22}, v_{23}, v_{47}, v_{46}, v_{15} and v_7, respectively.

Table S1. Assignments and local coordinates of red blood cells (RBCs) (21, 38, 39).

**Wavenumber (cm^{-1})**	**Assignment**
1637	v_{10} $v(C_\alpha C_m)$asym
1627	$v_{c=c}$ $v(C_a=C_b)$
1604	v_{19} $v(C_\alpha C_m)$asym
1585	v_{37} $v(C_\alpha C_m)$asym
1559	v_2 $v(C_\beta C_\beta)$
1547	v_{11} $v(C_\beta C_\beta)$
1431	v_{28} $v(C_\alpha C_m)$sym or -CH2
1395	v_{20} v(pyr quarter-ring)
1372	v_4 v(pyr half-ring)sym
1342	v_{41} v(pyr half-ring)sym or -CH2
1306	v_{21} $\delta(C_m H)$
1245	v_{13}
1228	v_{13} or v_{42} $\delta(C_m H)$
1166	v_{30} v(pyr half-ring)asym
1132	v_{22} v(pyr half-ring)asym
1090	v_{23} $v(C_\beta C_1)$asym
1245	v_{47} $v(C_\beta C_1)$asym
1228	v_{46} δ(pyr deform)asym
1166	v_{15} v(pyr breathing)
1132	v_7 δ(pyr deform)sym

Raman background

The background spectrum from the glass slide compared with the spectra acquired from the normal red blood cell (Fig. S1).

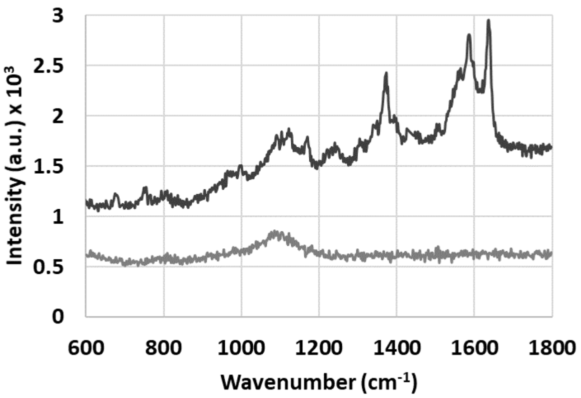

Figure S1. Spectra acquired from a normal red blood cell (upper) on the glass slide and from the glass slide alone (lower).

White blood cells and platelets

There were no significant differences in white blood cell counts (neutrophils, lymphocytes, monocytes, eosinophils and basophils) between high-dose control and experimental groups (Fig. S2). Similarly, platelet count and MPV do not differ (Fig. S3).

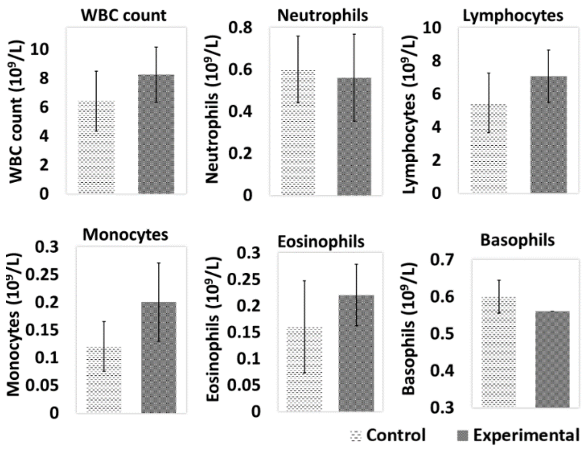

Figure S2. White blood cell (WBC) count, neutrophils, lymphocytes, monocytes, eosinophils and basophils for high dose control (n=5) and experimental groups (n=5). Data is presented as mean and standard deviation.

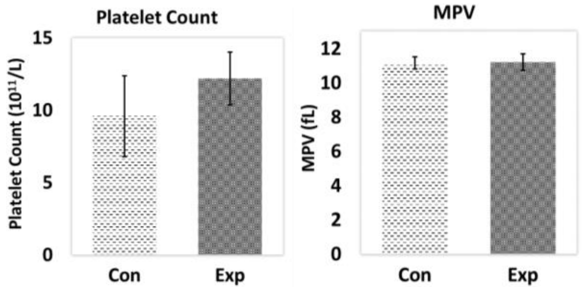

Figure S3. Platelet count and mean platelet volume (MPV) for high dose control (n=5) and experimental groups (n=5). Data is presented as mean and standard deviation.

Low-dose and high-dose comparison

Raman spectra from low-dose and high-dose group of normal-looking RBC, acanthocytes and echinocytes were compared. The low-dose Raman spectrum is from the manuscript and averaged across multiple cells of the same cell type, while the high-dose Raman spectrum is from one cell of each type. There were no significant differences between the two doses (Fig. S4).

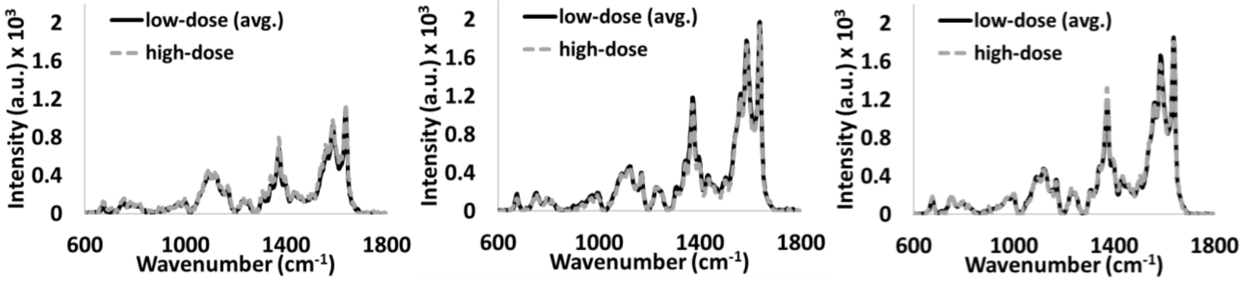

Figure S4. Raman spectra from normal-looking (left), acanthocytes (middle) and echinocytes (right) in the low and high dose groups. The low-dose spectra are those presented in Fig. 3. The high-dose spectra are single-cell Raman measurements from three RBCs in a high-dose subject.

Principal components and linear discriminant analyses

A classification analysis based on principal components analysis (PCA) and linear discriminant analysis (LDA) was performed and is shown in Fig. S5. Overlap between the classification of normal and normal-looking RBC, acanthocytes and echinocytes was observed. As expected, there was little overlap between the classification of poikilocytes and non-poikilocytes (normal and normal-looking).

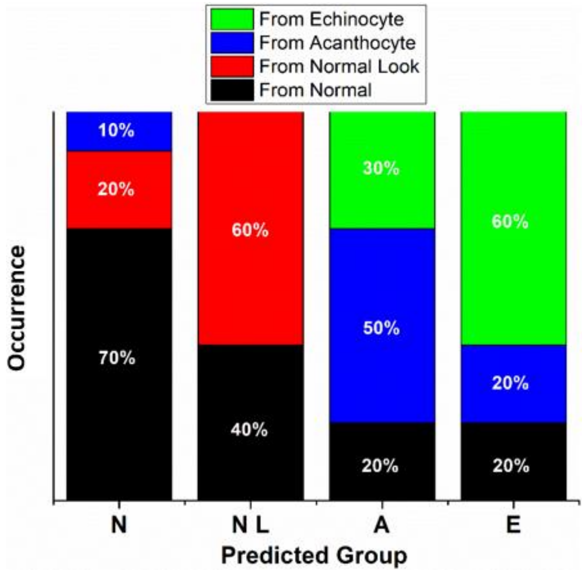

Figure S5. PCA-LDA analysis of 4 types of red blood cells: normal (N), normal-looking (NL), acanthocytes (A), and echinocytes (E). There was considerable overlap between the classifications of normal and normal-looking cells. Similarly, there was overlap between acanthocytes and echinocytes, the two types of poikilocytes studied.
